# A framework for indirect elicitation of the public health impact of gambling problems

**DOI:** 10.1186/s12889-020-09813-z

**Published:** 2020-11-16

**Authors:** Matthew Browne, Vijay Rawat, Philip Newall, Stephen Begg, Matthew Rockloff, Nerilee Hing

**Affiliations:** 1 School of Health, Medical & Applied Sciences, Central Queensland University, University Dr, Branyan QLD, Bundaberg, 4670 Australia; 2 La Trobe Rural Health School, La Trobe University, Bendigo, Australia

**Keywords:** Gambling, Gambling harm, Gambling problems, Elicitation, Health utility, Disability weights, Global burden of disease

## Abstract

Gambling problems are increasingly understood as a health-related condition, with harms from excessive time and money expenditure contributing to significant population morbidity. In many countries, the prevalence of gambling problems is known with some precision. However, the true severity of gambling problems in terms of their impact on health and wellbeing is the subject of ongoing debate. We firstly review recent research that has attempted to estimate harm from gambling, including studies that estimate disability weights using direct elicitation. Limitations of prior approaches are discussed, most notably potential inflation due to non-independent comorbidity with other substance use and mental health conditions, and potential biases in the subjective attribution of morbidity to gambling. An alternative indirect elicitation approach is outlined, and a conceptual framework for its application to gambling is provided. Significant risk factors for propensity to develop gambling problems are enumerated, and relative risks for comorbidities are calculated from recent meta-analyses and reviews. Indirect elicitation provides a promising alternative framework for assessing the causal link between gambling problems and morbidity. This approach requires implementation of propensity score matching to estimate the counterfactual, and demands high quality information of risk factors and comorbid conditions, in order to estimate the unique contribution of gambling problems. Gambling harm is best understood as a decrement to health utility. However, achieving consensus on the severity of gambling problems requires triangulation of results from multiple methodologies. Indirect elicitation with propensity score matching and accounting for comorbidities would provide an important step towards full integration of gambling within a public health paradigm.

## Background

In Australia and internationally, government agencies and statutory authorities have an expressed goal to minimise gambling-related harm [1, 2]. Likewise, the gambling research community has largely embraced a public health approach in which risks from gambling are understood primarily in terms of the harmful impacts of the behaviour [3–8]. This is distinct from pathological or models that approach harm indirectly in terms of the presumed financial or human cost among those dealing with a gambling disorder [9, 10], or in terms of a financial accounting of the costs attributable to problematic gambling [11].

There are clear advantages to understanding and managing gambling from a harm minimisation approach that have been discussed in detail elsewhere. However, the shift to a harm-centric model has not been without controversy, and raises important conceptual and practical questions regarding what exactly is meant by being ‘harmed by gambling’, and how this concept is to be measured [12–14]. Notably, this is a question that is also being addressed for other issues such as internet gaming disorder (IGD) [15]. On the one hand, there appears to be a good consensus on the spectrum of outcomes that are indicative of gambling-related harm. For example, a 72-item checklist of harms identified by Browne et al. [16] has been widely accepted, and covers a range of outcomes identified by other research [8, 17]. It is also broadly accepted that in the case of gambling, the primary mechanism for producing harm is the excessive financial losses incurred, and to a somewhat lessor extent excessive time spent gambling [18].

In contrast, there is some debate regarding the interpretation of severity and life-impact associated with varying profiles of gambling harm. Although there are several measures of gambling harm mentioned in the literature, to our knowledge, psychometric validation has only been reported for one measure: the Short Gambling Harms Scale (SGHS) [19]. SGHS scores appear to have a linear negative relationship with self-reported wellbeing. However, the SGHS is not universally accepted. Given that it includes several milder harms, such as ‘reduction of my savings’, Delfabbro and King [14] suggest that these might be rational opportunity costs, given the presumed recreational benefits of gambling, and therefore might not be true harms at all. Another criticism of self-report measures for gambling harms is that respondents might over-attribute life problems to gambling, leading to an upward bias – and making the social cost of gambling appear larger than it actually is [13, 14]. Alternatively, social desirability bias is another factor which may also affect reporting rates.

Given these critiques, it is worth emphasising that prior estimates of the scope and extent of gambling-related harm do not depend on the assumption that all items in measures such as the SGHS reflect significant amounts of harm. Indeed, the so-called ‘burden of gambling harm’ studies in Victoria [16] and New Zealand [20] were published before the development of the SGHS and followed established public health protocols for the assessment of the relative impact caused by a diverse range of conditions [21]. This involves, in broad terms, determining the typical symptomatology associated with the conditions, and then conducting direct comparisons between health conditions by community members and experts, regarding their relative impact on a person’s health. These relative comparisons between conditions, as well as more formal elicitation methods such as the Time Trade Off (TTO) task, are standard methods employed by research teams implementing the Global Burden of Disease (GBD) framework [21, 22], an integrated assessment framework introduced by Murray and Lopez [23] in the early 1990s to measure the global impact of a diverse range of conditions important to public health. As well as physical health-related conditions, the GBD also includes addictive behaviours (e.g., alcohol use disorder and drug use disorder), and mental health conditions (e.g., depression). Importantly vignettes employed for the burden of harm studies were constructed using neutral language, from surveys of gambling harms reported by different at-risk groups, thus reflecting the reported experiences of individual gamblers. Subsequently, people judging these vignettes – including gamblers, non-gamblers and experts – were free to ignore “opportunity costs” or any other harm that they considered to be insignificant when making their determinations about the severity of each person’s experience.

An important finding from the burden of gambling harm studies was that the majority of aggregate harm accrues to low and moderate risk gamblers as described by the Problem Gambling Severity Index (PGSI) categories. In attempting to confirm this finding, one alternative is to ask affected gamblers about their overall quality of their life, and empirically estimate the decrement in subjective wellbeing associated with increasing gambling problem severity. This is a standard technique known as ‘indirect elicitation’ and is commonly used in GBD studies. An analysis using the Australian Unity Wellbeing Index as a dependent measure, and controlling for potential confounding effects of multiple covariates, found nearly identical results as the burden of harm studies [24]. Another alternative is to treat harms as outcomes of interest, and count the number arising from different risk groups [25, 26]. However, this ‘harm counting’ approach is arguably too simplistic for assessing the true degree to which people in different risk categories are harmed by gambling, because of the considerable co-occurrence of many of these harms (e.g., relationship harms are coincident with emotional harms). Co-occurring harms might serve as a good indicator or reflection of an underlying continuum of harmful consequences but unless they are selected extremely carefully, they are unlikely to represent an exact linear composite of that construct [27]. In particular, problem gamblers are likely to experience a proliferation of harmful consequences that overlap in terms of their total contribution to a decrement in overall wellbeing.

As the brief discussion above suggests, an accurate picture of the scale and extent of gambling-related harm depends on a careful conceptual and measurement framework that links measures obtained from self-report scales to a recognised index of individual impact. As noted already, the burden of gambling harm studies [16, 20] implemented direct elicitation methods consistent with the GBD evaluation program. However, limitations acknowledged in these reports were that they did not attempt to consider any positive benefits that may co-occur with gambling harms, especially less severe harms, or control for the possible confounding effect of comorbid conditions. The latter has the most potential to be problematic, given the high degree of co-morbidity of gambling problems with other mental health and substance use disorders [28]. The present article will consider the scope for new alternative *indirect* elicitation approaches, also employed within the GBD assessment efforts, which may provide a useful means to address these and other concerns. While direct assessment methods use vignettes or descriptions of the experience of harmed gamblers, indirect assessment relies on statistical associations between gambling conditions (e.g., low-risk, moderate-risk and problem gambling) and the outcome of decrements to wellbeing.

The present article will provide an overview of the theoretical and methodological issues involved in the indirect assessment of gambling-related harm, including the attribution of causality to gambling, handling covariates and confounding variables, and gambling as a risk factor for other conditions that are harmful. It begins with the definition of gambling-related harm, and what that implies for measurement of this construct.

### A decrease in health and wellbeing caused by gambling

Several definitions of gambling-related harm exist. However, they consistently describe it explicitly as an adverse impact on health and wellbeing. For example, research funded by the UK Gambling Commission defines harm as “*the adverse impacts from gambling on the health and wellbeing of individuals, families, communities and society*” [8]. In Australia, the definition adopted by the Victorian Responsible Gambling Foundation includes a similar phrase, “*an engagement with gambling that leads to a decrement to the health or wellbeing of an individual, family unit, community or population*” [29]. These definitions are consistent with the World Health Organization’s (WHO) (1946) definition of health as “*a state of complete physical, mental and social well-being and not merely the absence of disease or infirmity*” [30]. In other words, harmful gambling describes the situation where a person’s health and wellbeing decreases as a consequence of their own, or someone else’s gambling.

From the above, there is no reason why harmful gambling cannot be placed in the same class as any other behavioural risk-factor that is determinantal to health and wellbeing, such as smoking, problematic alcohol and recreational drug use, or intimate partner violence. And like other behavioural risk-factors, a decrement to health and well-being from harmful gambling can be understood as accruing not just from non-fatal causes of ill-health but from fatal causes as well. Thus, the impact of harmful gambling can be assessed using the GBD framework in which of years of life lost to morbidity and mortality are both accounted for when quantifying the overall burden of disease.

### Screening for gambling harm

Common screens for gambling-related harm focus on items that capture common adverse consequences. Harm is also often confusingly subsumed under the more general construct of gambling problems [31]. Several items from the PGSI [32], for instance, arguably probe harmful consequences from gambling whereas others are symptoms of an underlying mental health condition without being necessarily harmful. There have also been efforts to specifically assess gambling harm apart from symptoms of a gambling disorder [33, 34]. However, to our knowledge, the only dedicated measure of gambling harm with published psychometric validation is the Short Gambling Harms Screen (SGHS) [19]. The SGHS was shown to be a highly reliable proxy for the comprehensive 72-item harm checklist, and therefore an appropriate measure of an underlying construct of being harmed by gambling.

There is controversy regarding how screens for harm should be interpreted, particularly with regard to lower levels of severity. For example, although there is consensus that PGSI-classified ‘problem gamblers’ (PG) are significantly harmed, there is not yet consensus on the degree to which ‘low risk’ (LR) or ‘moderate risk’ (MR) gamblers are harmed, or even if they are likely to be harmed at all. LR and MR gambling status has been associated with progressively greater decrements to subjective wellbeing, which supports the contention that they may have suffered harm [24]. Although the SGHS is also linearly associated with decreases in self-reported wellbeing, some doubts have been expressed as to whether lower scores on the SGHS are truly indicative of harm [13].

To summarise, several screens for gambling harm exist, they include similar content, and harm measures have been shown to have a relatively simple unidimensional structure. However, although screens for gambling harm and problems have been shown to be associated with a loss of wellbeing, they have – at most – been only partially assessed using formal health-epidemiological procedures, which we will discuss in more detail below.

### Capturing harm via disability weights

As mentioned in the introductory section, the GBD framework seeks to measure the global impact of a diverse range of conditions important to public health. At the centre of this framework are *disability weights* (DW), which aim to capture the average health loss associated with living with a particular manifestation of a condition. When combined with a measure of disease frequency in the population, this provides an estimate of the non-fatal burden of the condition in Years Lived with Disability (YLD). The fatal burden is measured in Years of Life Lost (YLL) and the total burden in Disability Adjusted Life Years (DALYs).

Disability weights for a range of substance use and mental disorders are included in this framework [22]. Disability weights are bounded between zero and one, with values close to zero having a negligible impact on health, and values close to one reflecting a profound impact making life intolerable. The reverse of this scale is often referred to as a Health State Valuation (HSV) as used in Quality Adjusted Life Years (QALYs), which are considered a cornerstone of health-economic analysis [35]. Although gambling disorder is included in the DSM-V and ICD-10, and the field itself considers gambling-related problems to be a public health issue [6], it is not currently evaluated in the GBD [36]. In contrast, the latest version of the GBD includes four different severity categories for alcohol use disorder, ranging from very mild (DW = 0.12) to severe (DW = 0.57) [36].

#### Direct elicitation

Disability weights estimated within the GBD and other disease burden studies are commonly done via elicitation methods. The GBD 2013 program relied heavily on a Discrete Choice Evaluation (DCE) protocol of *direct elicitation*, in which participants in a general survey were asked to compare pairs of lay-person condition descriptions, and indicate which condition was worse. Another common direct elicitation method is the time trade-off (TTO), which measures the extent to which respondents would be willing to give up an amount of life time to avoid a hypothetical condition and be in full health [37].

To our knowledge, the only applications to-date of direct elicitation to assess the impact of harmful gambling were undertaken in Australia [16] and New Zealand [20]. In these studies, both relative comparisons with other conditions, as well as TTO elicitation techniques, were used to assess condition descriptions reported by individuals at different levels of the PGSI [38]. The Australian study yielded DW of 0.14 for so-called low-risk gamblers, 0.29 for moderate-risk, and 0.46 for problem gamblers, which happens to correspond quite closely to DW estimated for the three lower levels of severity for alcohol use disorder within the GBD framework [36].

Direct elicitation methods that involve general population samples do not necessarily assume that the consensus evaluation is perfectly unbiased. Rather, they assume that because it is the public that is affected by health-related policies, then the public’s view regarding the impact of conditions is the most valid and meaningful. To illustrate, if the consensus community view is that the severity of gambling problems is similar in magnitude to that of alcohol abuse, and it is the community that bears the costs and benefits of both behaviours, then there are few technical or theoretical grounds with which to challenge that evaluation. Nevertheless, the aforementioned Australian and New Zealand burden of harm studies that used the direct elicitation method to find DWs for gambling also included a panel of experts from the fields of gambling research and treatment. Interestingly, there was broad concordance between the results of these experts and public views.

These observations notwithstanding, gambling may present special difficulties when it comes to *attribution* of symptoms or harm to the condition of harmful or disordered gambling – a topic that will be discussed in detail below. Furthermore, it should be noted that the vast majority of the disease burden estimates mentioned above incorporate disability weights that assume conditions occur in isolation, and are therefore vulnerable to the problems that arise due to comorbidity [39]. This is indeed also true for the ‘burden of harm’ estimates accomplished for gambling [4]. Although some novel approaches have been developed to overcome this problem, they necessarily involve some simplifying assumptions, as will be discussed below.

#### Comorbidity

Comorbidity, for the purposes of this discussion, describes the situation where two or more health problems occur in a person simultaneously, either by chance or because the conditions are related to each other in some way. *Independent* comorbidity is where the probability of having multiple conditions at the same time equals the product of the probabilities for each condition. *Dependent* comorbidity, on the other hand, is where the probability of having multiple conditions is greater than the product of the probabilities for each condition, and occurs because of common causal pathways (for example common risk factors causing both diabetes and heart disease) or because one health problem may increase the risk of another.

Both types of comorbidity can be problematic for the conceptual framework proposed by Murray and Lopez [23], particularly when the set of available disability weights is comprised of evaluations for each health state as it occurs independently from others. As noted above, this includes the vast majority of burden of disease studies to date.

The severity of a health state associated with two or more conditions in combination may not necessarily be the sum of the disability weights for each condition. In most cases, it is likely to be less than the sum. In others, there may be exacerbating effects on overall health of having the combination of conditions. For example, the experience of symptomatic grade 2 osteoarthritis of the hip and severe vision loss together is probably not as disabling as the addition of the two weights for these health states (0.14 and 0.43, respectively). However, the experience of the latter with profound deafness may be equal to or even more disabling than the simple summation approach indicates.

In an early response to this problem, Mathers et al. [40] proposed an adjustment that assumed health state valuations (that is, 1 minus the disability weight) are multiplicative, so that a combined weight for two conditions is more severe than the weight for either condition on its own but less than if the weights were simply added together. In this approach, the combined severity weight for causes *k* = 1 and *k* = 2 is given by,

1 $$ {DW}_{\left[1,2\right]}=1-\left(1-{DW}_1\right)\times \left(1-{DW}_2\right) $$

This can be generalised to *n* conditions thus,

2 $$ {DW}_{\left[1,n\right]}=1-\prod \limits_{j=1}^n\left(1-{DW}_j\right) $$

where () denotes the product operator.

To illustrate, if an individual was experiencing both severe alcohol use disorder and is also classified as a problem gambler, the combined DW is not 0.57 + 0.46 = 1.03, but rather 1 - (1–0.46)*(1–0.57) = 0.77.

Equation (2) has been extensively used to derive combined weights for comorbid conditions in subsequent applications of the framework. Work by Flanagan et al. [41] indicates that, in the absence of anything else, the multiplicative approach to deriving composite weights is reasonably robust.

Mathers’ initial implementation derived individual weights consistent with these composite weights by leaving the weight for the most severe condition unchanged but adjusting the weight for the milder condition such that it equalled the composite weight minus the weight for the more severe condition. Implicit in this approach is an assumption that the prevalence of a set of comorbid conditions is equal to the product of the individual prevalences of these conditions; in other words, that health problems occur independently of each other (see [42]). Subsequent work demonstrates that correcting for dependence between groups of conditions has a non-trivial impact on comorbidity-adjusted disability weights and ultimately integrated measures such as DALYs [43].

The difficulty associated with controlling for comorbidity arises due to the perceived impracticality of obtaining empirical data regarding comorbidity rates and effects on DWs for every possible combination of conditions included in a typical GBD analysis [44]. However, as demonstrated by Gadermann et al. [45] for 19 comorbid mental and chronic physical disorders at least, it is possible to gather self-report data on multimorbidity, as well health status, and then to model the simultaneous main and interactive effects of each condition on health. Importantly, rather than relying on DWs elicited directly from evaluations of health states (described above), this kind of approach infers the DW attributable to a given condition based on the self-reported health states of affected individuals. This is sometimes referred to as *indirect elicitation* of DW. In this calculation, rather than assessing “how bad” suffering is from each condition, instead a person’s overall health and wellbeing is assessed, and a statistical association is made, usually by means of regression, between a person’s poor health and the presence of a wide range of disease conditions. From this association, DWs can be indirectly inferred in a sample from the strength of each association between a given disease and people’s measured general health and wellbeing.

#### The challenge of attribution

Given the ubiquity of comorbidity in a population, and the multiple simultaneous effects on health and wellbeing that this gives rise to, a key requirement of DW estimation is to be able to confidently attribute an impact on health to a given condition. As mentioned above, directly elicited DWs can be scaled given the presence of multiple conditions using mathematical heuristics, or alternatively, modified empirically from observed interaction effects. However, even in the absence of comorbidity, the attribution of causality is still a problem for integrated health assessment techniques such as the Murray and Lopez [23] framework, given that the necessary empirical data are rarely available.

To summarise, a direct elicitation approach relies on the ability of either experts or community members to:

- Describe the symptomatology that occurs as a result of having a condition; i.e. forming condition descriptions
- Assess the total impact of that symptomatology, relative to a healthy individual.

Thus, this approach entails that the task of attribution is delegated to participants, community members, or experts via elicitation protocols. On the one hand, this is preferable to researchers making arbitrary judgements regarding symptomology or severity. On the other hand, there is the possibility that both the participants and the elicitation methods themselves may introduce various forms of bias. Investigation and resolution of these issues are areas of ongoing methodological research [46–49].

To conclude, direct elicitation of DW is the current standard within the GBD framework to assess burden of disease. The elicitation techniques employed for gambling to date have followed the same principles and methods used for other conditions, including harmful alcohol use. These ‘raw’ DWs can then be scaled to account for comorbidity when calculating YLD within an integrated assessment framework. However, adjusting for comorbidity is often done via an analytic formula, rather than based on empirical data. Furthermore, integrated frameworks often do not take into account dependent comorbidity, and therefore may apply insufficient adjustments for highly comorbid conditions like gambling problems [50]. For instance, Petry and colleagues [51] have estimated that 73.2% of people in the United States with a gambling problem also have an alcohol use disorder, which should strongly affect proper DW adjustment for both conditions. Finally, direct elicitation generally requires evaluations of a vignette or condition description made by third parties. Thus, it is not ‘direct’ in sense of eliciting health state information directly from the affected individuals, which might compromise its validity.

#### Indirect elicitation

Indirect elicitation is ‘indirect’ in the sense that condition descriptions are not evaluated directly for their impact. Rather, individuals suffering from the condition are compared to those who do not have the condition in terms of their self-reported HSV or health and wellbeing. Self-reported HSVs can be elicited using a Visual Analog Scale (VAS) [45] or a survey instrument such as the SF-12 [52]. It is important to recognise that the goal is to estimate the presumed causal effect of a condition on HSVs from cross-sectional data. This is similar to estimating the HSV under a counterfactual scenario in which the condition was eliminated [53]. In this scenario, the challenge of attribution is not relegated to the judgements of participants, but rather made the subject of statistical analysis. To make plausible inference of causality, such studies must take great care to accomplish two goals:

1. To estimate a *propensity model* (see [54]) - the function of risk factors that lead some individuals to have the condition when others do not.
2. To estimate a *causal model* (see [55]) – the unique effect of the condition on HSV after controlling for comorbid health-related issues.

The same covariates may appear in both the propensity model and the causal model. The propensity model is used to match the control group and the condition group as closely as possible, which may involve both purposeful sampling and case weighting [53–56]. That is, the purpose of the propensity model is to find a matched sample of others not suffering from gambling problems or harm who otherwise resemble the gambling-harmed participants on key risk factors. In the case of gambling, the causal model should incorporate known comorbidities (e.g. alcohol misuse) that are also known to affect wellbeing, to avoid attributing non-gambling impacts (e.g. those due to alcohol) to gambling.

Despite the challenges involved, indirect elicitation studies complement direct elicitation studies in several important ways. First, they are based on HSVs elicited from the individuals suffering from the condition, arguably increasing their validity. The respondents are *not* asked to attribute the degree to which their health was affected by a given condition, which eliminates a potential source of bias by virtue of people over (or under) attributing the contribution of the condition to their wellbeing. Also, they provide the opportunity to gather detailed comorbidity information, thus providing empirical estimates of both dependent and independent comorbidity rates and consequently the information needed to adjust DWs for these comorbidities. Finally, because self-reported HSV can be influenced by positive or negative effects of a behaviour, there is no implicit assumption that gambling can only have a negative impact on wellbeing. Any positive contributions to wellbeing, such as those measured by Rockloff et al. [57], will be balanced against negative contributions, which eliminates another important source of bias.

### Indirect elicitation for evaluating harm from gambling

The factors that are associated with gambling problems are well understood. It is also known that gambling problems are also highly comorbid with other addictive and mental health disorders. Thus, the indirect elicitation method via self-reported HSVs, combined with a propensity score weighting framework, presents particular benefits in evaluating the effects of gambling-related harm. It provides a means to appropriately adjust DWs, while also implicitly recognising any potentially positive contributions of gambling to health and wellbeing that may partially offset the negatives. Direct elicitation, while having its own strengths, has neither of these benefits.

**Fig. 1:**
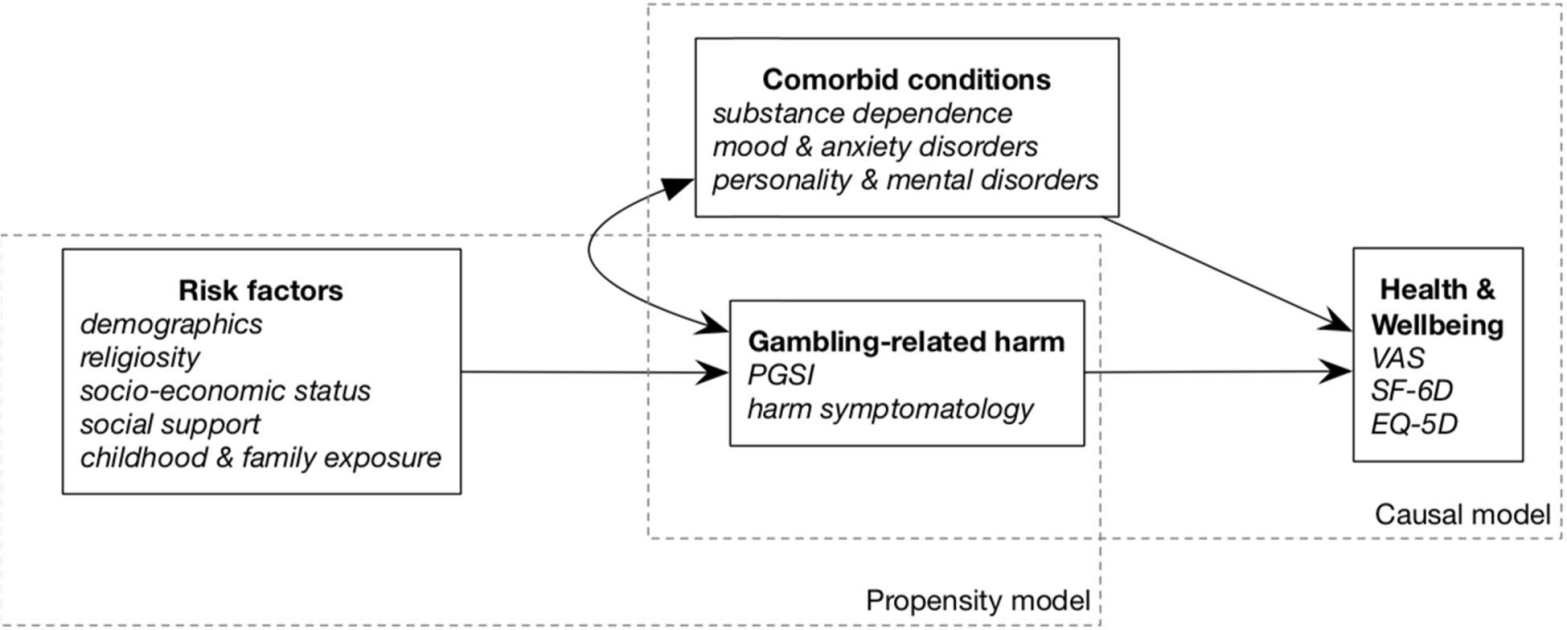
Framework for propensity score matching and causal inference for health-related impacts of gambling

Figure 1 illustrates the basic framework for this kind of evaluation applied to gambling-related harm. Two statistical models are involved. First, the propensity model describes the effect of risk factors on the likelihood of experiencing gambling-related harm. In practical terms, that comparisons between harmed and unharmed individuals are matched and weighted as much as possible with respect to risk factors. For example, if the majority of problem gamblers are young men, then it is most appropriate to compare them with a control group that has a similar preponderance of young men. Conceptually, this stage requires a discrete approach to categorising individuals into case and control groups. An accepted population screen such as the PGSI may be used for this purpose.^1^ Second, the causal model links indices of gambling harm with HSVs. This requires accounting for comorbid and non-independent conditions in the regression model, to avoid attributing common variance exclusively to gambling. Discounting effects due to comorbidity can be handled via (negative) interaction terms between conditions. Such estimates can ultimately feed into a fully integrated computational analysis for YLDs or DALYs such as the GBD.

As this framework makes clear, indirect elicitation of the health impacts of gambling depend not only on good indices of gambling harm and HSVs, but also on good knowledge of both risk factors and co-morbid health-related conditions with harmful gambling. Thus, we will now review and summarise the current knowledge regarding these two sets of covariates.

#### Risk factors for gambling problems

This section enumerates risk factors for gambling problems (i.e. disordered and/or harmful gambling) which have been identified in previous research and should be considered for inclusion in an indirect evaluation framework. Sources considered included meta-analyses, systematic reviews, theoretical models, and recent original research that has not been included in the aforementioned sources (Table 1). To our knowledge, this list includes all significant relevant systematic reviews on this topic.

**Table 1:** Key sources examined to identify risk factors for gambling problems

Reference	Study design
Abbott et al. [58]	Systematic review
Browne et al. [59]	Original research
Cunha et al. [60]	Original research
Dowling et al. [61]	Systematic review & Meta-analysis
Hing et al. [62]	Original research
Johansson et al. [63]	Systematic review
Miller [64]	Systematic review
Sharpe [65]	Theoretical model
Sharpe & Tarrier [66]	Theoretical model
Vasiliadis et al. [67]	Systematic review
Williams et al. [68]	Systematic review

The risk factors identified from these sources were grouped into five broad categories: childhood/family, cultural, demographic, geographic, and personal. The goal was to identify the most important risk factors that represent unique factors that predict whether an individual is likely to experience gambling problems.

Previous research has highlighted issues surrounding a person’s childhood and family upbringing, often under different and yet conceptually similar terms. Childhood gambling exposure via parental gambling problems or children participating in parental gambling activities have been identified [59]. That is, the extent to which parents gamble may drive exposure to gambling during childhood, which has been associated with an increased risk of becoming a problem gambler in adulthood [58, 59, 68]. Family structure more broadly is also a childhood risk factor, with single parent households [64], and lower levels of parental supervision [61] being found to be risk factors for adult gambling problems. Child-specific factors have also been highlighted, for example the child’s antisocial behaviours [61], and childhood ADHD (Attention deficit hyperactivity disorder) [65, 66]. However, rather than including recalled childhood ADHD in the model, it may be better to include adult impulsivity, which is also highlighted below. Adult impulsivity may be more reliably measured and strongly correlated with childhood ADHD. Children are also highly affected by their peer group, and peer antisocial behaviours have therefore been highlighted as a risk factor [61], as has peer gambling [68]. Thus, a propensity model for gambling problems ought to include variables assessing childhood family structure, as well as childhood gambling or childhood exposure to gambling from family and peers. These are all fairly objective factors, that should be recalled with less noise, and be highly correlated with, other factors such as childhood antisocial behaviors.

Cultural factors also appear relevant to adult gambling problems. Ethnic minority groups have been shown to be at a greater risk of adult gambling problems [63], as well as people who speak a language other than English at home [62], and those born overseas [58, 63]. Religiosity has also been highlighted as a risk factor [58, 59], although this did not appear as a statistically significant factor in a recent meta-analysis [61].

A large number of demographic factors have been shown to be predictors of adult gambling problems. Poor academic performance [61] and lower educational attainment [59, 60, 68] are commonly identified risk factors. Male gender is as another key risk factor, with young men being several times more likely to experience problems than many other age-gender categories [58, 60, 61, 63]. Young women, as well as men, are generally more likely to experience problems [59, 62, 63]. Unemployment also presents a risk [62, 63], and occupational status more broadly appears related to whether or not one experiences problems [58, 59, 64]. Relatedly, high income broadly appears to be a relevant protective factor [58, 59, 68], as is high socio-economic status [58, 61, 64]. Marital status is also relevant, with both people who are currently single [58, 59], and those who are divorced [64] at higher risk compared to others. These findings accord with results regarding household living status, with those living alone [64] or in a group household [62] considered to be at higher risk. In general, it is clear that being in a more vulnerable social and socioeconomic situation is a key dimension which predicts whether or not one will experience problems with gambling. A variety of brief indices could be used to capture this information for propensity matching and risk modelling, including age, gender, employment status, income, education, and marital status.

Geographic and environmental factors also appear relevant to the development of gambling problems. Living in a large city has been identified as a risk factor [58, 63], as well as the distance between one’s residence and the gambling venue [59]. More generally, the availability of gambling products represents an inherent risk [65, 68], which in the contemporary context, represents a combination of both land-based and remote (online and mobile) gambling opportunities. However, the availability of gambling products is a complex and dynamic risk factor, which the field is still in the process of addressing [67]. Nevertheless, at a minimum, measures of venue distance and metropolitan / regional / rural residential location should be included in a propensity model.

Finally, stable personal or psychological factors also appear relevant to the risk of developing gambling problems. A number of psychological factors, such as personality disorders or substance dependencies are more appropriately considered under the label of comorbidities. Impulsivity, however, is reliably observed as an important risk factor [61, 63, 65, 66, 68], and emerged as the single strongest correlate of adult gambling harm in one study [59]. In terms of propensity matching, adult impulsivity may be a more reliable construct to match the two groups on than the related traits of childhood ADHD [65, 66] or childhood antisocial behaviours [61]. The similar construct of sensation seeking has also been consistently highlighted as a risk factor for gambling problems [58, 61, 63]. The experience of early big gambling wins is thought to be an important risk factor [65], but did not emerge as a statistically significant predictor in a recent meta-analysis [61]. Trait impulsivity and/or sensation seeking are therefore the most relevant psychological traits to consider for propensity modelling.

In practical terms, for the purpose of generating a suitable propensity model for gambling problems, it is not necessary to include an exhaustive list of all correlates. As demonstrated by Browne et al. [59], many risk factors are correlated with each other, and therefore do not necessarily provide unique information in a simultaneous regression model. Furthermore, it is not necessary for the propensity model to be ‘perfect’. Rather, the goal is to ensure that a good case can be made for gambling, rather than some other variable(s), have an instrumental role in explaining differences between the case and control groups [53].

#### Health conditions comorbid with gambling

Gambling disorders are known to have significant co-morbidities with other psychiatric disorders [28]. High rates of problem gamblers have also been observed in mental health settings, with a recent study reporting rates of problem gambling among patients eight times that observed in the general community [69]. While the extent of comorbidities with gambling problems has been well documented narratively [70], arguably the following three research articles provide the strongest evidence for understanding these co-morbidities in both clinical and community samples:

1. Dowling et al. [71]: A systematic review and meta-analysis for the prevalence of co-morbid psychiatric disorders among treatment-seeking problem (including pathological) gamblers
2. Lorains et al. [72]: A systematic review and meta-analysis for the prevalence of co-morbid disorders in population representative surveys of problem (including pathological) gamblers
3. Dowling et al. [73]: A systematic review and meta-analysis for the prevalence of co-morbid personality disorders among treatment seeking problem (including pathological) gamblers

Table 2 below explains the constituent parts of Table 3. Results drawn from the three aforementioned reviews form part of Table 3 (specifically columns 2 & 3). Table 3 also contains information drawn from a range of other sources and was generated to enable a better understanding of comorbidities and relative risk, which was not made explicit in the cited articles.

**Table 2:** Column guide

Column	Description
1. Disorder	The specific comorbid disorder
2. Number of estimates & 3. Mean comorbid prevalence (%)	These two figures/columns should be interpreted in conjunction. Column 2 is a count of *individual estimates* that were used to estimate the mean prevalence of co-morbidity. For example, the *mean co-morbid prevalence* of ‘alcohol abuse’ was derived using nine studies. Column 3 is the actual *mean comorbid prevalence*. For example, the co-morbidity of ‘alcohol abuse’ among problem gamblers was estimated to be 18.2%. The figures in this column were derived from Dowling et al. [71, 73] who examined treatment seeking problem gamblers, and Lorrains et al. [72] who examined community samples of problem gamblers.
4. Community prevalence (%)	This figure is the rate of the disorder observed in the *general population irrespective of problem gambling status* (e.g. 8.5% community prevalence for any alcohol use disorder)
5. RR (*SE*)	Relative risk (RR) is the likelihood of having a specific co-morbid disorder for a problem gambler, compared to the general population. E.g. The rate of alcohol abuse is almost 4*x* higher among problem gamblers than in the general population. This calculation was based on estimates from previous research, and associated standard error (*SE*) rates are approximated by propagating uncertainty for both the numerator and denominator, using a first-order Taylor expansion ${\sigma}_f\approx \left|f\right|\sqrt{{\left(\frac{\sigma_A}{A}\right)}^2+{\left(\frac{\sigma_B}{B}\right)}^2-2\frac{\sigma_{AB}}{AB}}$ where $f=\frac{A}{B}$ and A and B represent the probability of a gambler and the general population to have the condition, respectively. We assume the covariance term to be zero.
6. DW	Disability weights (DW) quantify the health loss associated with an outcome and are measured on a scale from 0 (indicating full health) to 1 (a state equivalent to death) [36]
Other notes	• Where possible, 95% confidence intervals for estimates are presented in square brackets • A dash in any cell ‘-’ indicates that piece of information was not able to be obtained • The information was obtained from a wide range of sources. Due to methodological variations between studies (e.g. diagnostic tools used) the figures should be interpreted with caution when comparing. A discussion of these issues will follow later in this paper.

**Table 3:** Prevalence of comorbid disorders among problem/pathological gamblers

Disorder	Number of estimates	Mean comorbid prevalence (%)	Community prevalence (%)	RR (***SE***)	DW
Any DSM-IV Axis 1 disorder	5^a^	74.8 [36.5–93.9]	20.0 [18.9–21.0] [74]	3.7 (0.74)	–
*Any alcohol or substance use disorder*	10^a^	22.2 [16.1–29.8]	5.1 [4.5–5.8] [74]	7.8 (0.85)	–
	3^b^	57.5			
Any alcohol use disorder	12^a^	21.2 [5.6–28.1]	8.5 (*SE* = 0.24) [75]	2.9 (0.68)	Very mild (0.123) [36] Mild (0.235) [36] Moderate (0.373) [36] Severe (0.570) [36]
	8^b^	28.1			
Alcohol abuse	9^a^	18.2 [13.4–24.2]	4.7 (*SE* = 0.18) [75]	3.9 (0.60)	–
Alcohol dependence	7^a^	15.2 [10.2–22.0]	3.8 (*SE* = 0.14) [75]	4.0 (0.81)	–
Any substance (non-alcohol) use disorder	7^a^	7.0 [1.7–24.9]	2.0 *(SE =* 1.00) [75]	6.1 (4.23)	–
	3^b^	17.2			
Substance (non-alcohol) abuse	8^a^	6.6 [3.3–12.7]	1.4 (*SE* = 0.08) [75]	4.7 (1.73)	–
Substance (non-alcohol) dependence	6^a^	4.2 [1.5–11.4]	0.6 (*SE* = 0.05) [75]	7.0 (4.25)	–
Nicotine dependence	3^a^	56.4 [35.7–75.2]	12.8 (*SE =* 0.39) [75]	4.6 (0.80)	–
	4^b^	60.1			
Cannabis use disorder	3^a^	11.5 [4.8–25.0]	1.5 (*SE =* 0.08) [76]	7.7 (3.46)	Mild dependence (0.329) Moderate to severe (0.479) [36]
*Any mood disorder*	10^a^	23.1 [14.9–34.0]	9.2 (*SE* = 0.22) [75]	3.3 (0.54)	–
	3^b^	37.9			
Major depressive disorder	17^a^	29.9 [20.5–41.3]	7.1 (*SE* = 0.20) [75]	3.7 (0.75)	Mild (0.145) [36] Moderate (0.396) [36] Severe (0.658) [36]
	6^b^	23.1			
Dysthymic disorder	3^a^	6.7 [4.8–9.2]	1.8 (*SE* = 0.09) [75]	3.7 (0.65)	0.33–0.38 [77]
Bipolar disorder	10^a^	8.8 [4.4–17.1]	Mania 1.7 (*SE* = 0.08) [75]	5.5 (1.92)	Manic episode (0.492) Residual state (0.032) [36]
	6^b^	9.8			
*Any anxiety disorder*	10^a^	17.6 [10.8–27.3]	11.1 (*SE* = 0.33) [75]	2.5 (0.39)	Mild (0.030) [36] Moderate (0.133) [36] Severe (0.523) [36]
	3^b^	37.4			
Obsessive compulsive disorder (OCD)	7^a^	8.2 [3.4–18.6]	1.2 (*SE* = 0.30) [78]	6.8 (3.66)	0.12–0.60 [77]
Panic disorder	6^a^	13.7 [6.7–26.0]	Without agoraphobia 1.5 (*SE* = 0.07) [75]	9.1 (3.31)	0.11–0.69 [77]
Generalised anxiety disorder (GAD)	4^a^	14.4 [3.9–40.8]	2.1 (*SE* = 0.10) [75]	6.1 (4.49)	0.17–0.60 [77]
	3^b^	11.1			
Post-traumatic stress disorder (PTSD)	4^a^	12.3 [3.4–35.7]	4.7 (*SE* = 0.17) [79]	2.6 (1.76)	0.11–0.51 [77]
Social phobia	3^a^	14.9 [2.0–59.8]	2.8 (*SE* = 0.13) [75]	5.3 (5.27)	0.17–0.59 [77]
*Other disorders*					
Intermittent explosive disorder	3^a^	4.6 [2.5–8.4]	3.9 (*SE* = 0.30) [80]	1.2 (0.40)	–
Kleptomania	3^a^	2.7 [1.2–5.9]	0.4 [0.1–1.0] [81]	6.8 (4.90)	–
Psychotic disorder	5^a^	4.7 [3.4–6.5]	Psychosis 0.4 (*SE =* 0.1) [82]	11.8 (3.54)	Schizophrenia [36] Acute state (0.778) Residual state (0.588)
Somatoform disorder	5^a^	3.6 [1.6–8.0]	0.8 [0.3–1.4] [83]	4.5 (2.58)	0.144 [84]
Adjustment disorder	5^a^	9.2 [4.8–17.2]	0.3 [0.1–0.5] [85]	30.7 (14.83)	–
ADHD	4^a^	9.3 [4.1–19.6]	4.4 (*SE =* 0.6) [86]	2.1 (0.94)	0.045 [36]
Any personality disorder (PD)	9^c^	47.9 [29.8–66.7]	7.8 [6.1–9.5] [87]	6.1 (1.39)	–
*Any cluster A disorder*	4^c^	6.1 [1.5–22.1]	3.8 [3.2–4.4] [87]	1.6 (1.39)	–
Paranoid personality disorder	8^c^	10.1 [4.2–22.1]	2.3 [1.6–3.1] [87]	4.4 (2.12)	–
Schizoid personality disorder	8^c^	6.0 [2.5–13.7]	1.1 [0.7–1.5] [87]	5.5 (2.79)	–
Schizotypal personality disorder	7^c^	4.1 [0.8–19.4]	0.8 [0.5–1.1] [87]	5.1 (6.01)	–
*Any cluster B disorder*	4^c^	17.6 [6.0–41.8]	2.8 [1.8–3.7] [87]	6.3 (3.44)	–
Antisocial personality disorder	14^c^	14.0 [10.5–18.4]	1.4 [0.8–2.3] [87]	15.3 (4.42)	–
	2^b^	28.8			
Borderline personality disorder	8^c^	13.1 [4.3–33.5]	1.8 [1.2–2.5] [87]	7.3 (4.35)	0.193 [84]
Histrionic personality disorder	7^c^	6.3 [1.0–30.4]	0.6 [0.4–0.9] [87]	10.5 (12.7)	–
Narcissistic personality disorder	8^c^	16.6 [8.0–31.2]	1.9 [0.1–5.6] [87]	8.7 (7.16)	–
*Any cluster C disorder*	4^c^	12.6 [4.8–29.1]	5.0 [4.2–5.9] [87]	2.5 (1.26)	–
Avoidant personality disorder	6^c^	13.4 [5.9–27.5]	2.7 [1.9–3.7] [87]	5.0 (2.21)	–
Dependent personality disorder	8^c^	6.0 [1.4–22.5]	0.8 [0.5–1.3] [87]	7.5 (7.00)	–
Obsessive-compulsive personality disorder	6^c^	13.4 [5.9–27.5]	3.2 [2.4–4.1] [87]	4.2 (1.81)	–

*DSM-IV* Diagnostic and Statistical Manual of Mental Disorders - 4th edition

^a^ [71]; ^b^ [72]; ^c^ [73]

Table 3 highlights elevated rates for all disorders among problem (including pathological) gamblers compared to the general population. These disorders vary greatly in terms of their base-rate in the general population, and in terms of the increased risk of gamblers to have the disorder. Problem gamblers are almost four times more likely to have a comorbid mental disorder (Axis-I).

Problem gamblers are almost eight times more likely to also be experiencing alcohol or drug use disorders compared to the general population. The rate for alcohol use disorders is almost three times higher among problem gamblers, and six times higher for a drug use disorder. More specifically nicotine dependence (4.6*x*) and cannabis use disorder (7.7*x*) were significantly elevated among problem gamblers.

Mood disorders are over three times more common among problem gamblers. Problem gamblers are almost four times more likely to have co-occurring major depressive disorder or dysthymic disorder, and five times more likely for bipolar disorder.

Anxiety disorders are two and a half times more common among problem gamblers than in the general population. More specifically, panic disorder, obsessive compulsive disorder, and generalised anxiety disorder, are *each* over six times common among problem gamblers.

Problem gamblers are six times more likely to be diagnosed with a co-occurring personality disorder. While problem gamblers were at higher risk for all types of personality disorder, Cluster B disorders were particularly elevated (6.3*x*); with anti-social personality disorder (15.3*x*) and histrionic personality disorder (10.5*x*) being particularly prevalent among problem gamblers.

### Limitations

The information presented in Table 3 was obtained from a range of sources and in this task we were limited to the research available. The following points should be taken into account when interpreting the findings.

The studies examining rates of comorbidities among gambling populations across three meta-analyses [71–73] were largely from Western countries (particularly the US) and lacked broader cultural/geographical representation. To offset this limitation, where possible, we sourced community prevalence rates for comparison from similar countries.

Meta-analyses by virtue of combining estimates from a range of studies include a range of biases. Thus, the reported prevalence rates may be heterogeneous as a result of methodological differences such as sampling and use of diagnostic tools (see [71–73] for more details).

Estimates of comorbid condition prevalence vary in terms of whether they were derived from treatment-seeking problem gamblers [71, 73] or general population screens for problem gambling [72]. Given that treatment-seekers are likely to be on the most severe end of the spectrum, estimates of prevalence and relative risk are likely to be relatively larger for the former group. Likewise, the degree of relative risk can be assumed to be proportionately lower among low-risk and moderate-risk gamblers.

Our calculation of relative risk required sourcing the community prevalence for disorders. Given community prevalence rates presumably include problem gamblers, the RR may be slightly underestimated, due to problem gamblers forming part of the broader population. Furthermore, the rates of some disorders in the general community were not able to be sourced. For example, to our knowledge there are no reliable population estimates for kleptomania. Thus, we used estimates for kleptomania derived from a single sample of 791 college students.

Notwithstanding the above limitations, this collation of evidence provides a useful overview of which disorders are most strongly comorbid with gambling, along with population base rates and disability weight estimates where possible. Although the rate of mental health and substance use conditions is generally higher among problem gamblers, alcohol/substance use disorders, mood disorders, anxiety disorders, and personality disorders have the strongest degree of association. In a multivariate evaluation of the instrumental role of gambling in driving changes in health and wellbeing, it is not practical nor necessary to include every possible co-morbid health condition. Rather, it is desirable to include the more severe and more prevalent conditions, that demonstrate strong non-independent comorbidity with gambling problems.

## Conclusions

We have argued that gambling harm is best understood as a decrement to health and wellbeing. It follows that epidemiological tools designed to assess the impact of conditions on health have direct application to gambling, just as they do for similar addictive and behavioural problems. Two major studies in Australia and New Zealand have adopted a direct approach to assess the impact of gambling. This approach can be complemented by an indirect approach, that relies on self-reported HSVs, avoids potential biases in self-attribution of the impact of gambling, and takes into account comorbid health conditions. Both approaches have been employed successfully in the epidemiological literature on evaluating other addictive behaviours and mental health conditions, leading to their inclusion in frameworks for estimating their global burden on health. Unlike alcohol, substance, or intimate partner abuse, gambling problems are not yet included in frameworks such as the GBD. Arguably, this omission perpetuates a policy environment in which the benefits of gambling as a source of revenue to government are readily apparent, whilst the true public health impact of this behaviour remains largely invisible [2, 88].

Given that the field of gambling studies has embraced a public health approach for some decades now, the lack of progress in aligning the evaluation of the impacts of gambling with other risky health related behaviours is both striking and disappointing. The present review has provided a primer on epidemiological evaluation frameworks in terms of their potential application to gambling and outlined the case for supplementing prior efforts at direct elicitation with an indirect elicitation framework. We have summarised the state of knowledge regarding risk factors that determine propensity for the development of gambling harm, as well as comorbid conditions. This information is necessary for implementation of indirect elicitation of the health impacts of gambling-related harm. It is intended as a resource for research teams planning to evaluate gambling using counterfactual logic, using matched sampling propensity weighting, while also accounting for comorbid disorders. Our view is that present information on both sets of covariates is more than sufficient for indirect elicitation of the relationship between problematic gambling behaviour and health. Future research should focus on implementing this framework, thereby facilitating the integration of gambling within the GBD and similar public health assessment frameworks.

## Data Availability

All data generated or analysed during this study are included in this published article.

## Abbreviations

ADHD: Attention deficit hyperactivity disorder
DALY: Disability adjusted life year
DCE: Discrete choice evaluation
DSM-IV: Diagnostic and statistical manual of mental disorders - 4th edition
DSM-V: Diagnostic and statistical manual of mental disorders - 5th edition
DW: Disability weights
GAD: Generalised anxiety disorder
GBD: Global burden of disease
HSV: Health state valuation
ICD-10: International statistical classification of diseases and related health problems - 10th revision
IGD: Internet gaming disorder
LR: Low risk
MR: Moderate risk
OCD: Obsessive compulsive disorder
PD: Personality disorder
PG: Problem gambler
PGSI: Problem gambling severity index
PTSD: Post-traumatic stress disorder
QALY: Quality adjusted life year
RR: Relative risk
SE: Standard error
SGHS: Short gambling harms scale
TTO: Time trade off
UK: United Kingdom
VAS: Visual analogue scale
WHO: World Health Organization
YLD: Years lived with disability
YLL: Years of life lost

## Footnotes

1 It should be noted that the PGSI measures both problem-gambling symptoms and harmful outcomes, but for the purposes of this analysis the harmful outcomes are the measurement property of interest.

## References

[1] Costes J-M, Bowden-Jones H, Dickson C, Dunand C, Simon O (2019). A logical framework for the evaluation of a harm reduction policy for gambling. Harm reduction for gambling.

[2] Wardle H, Reith G, Langham E, Rogers RD (2019). Gambling and public health: we need policy action to prevent harm. BMJ..

[3] Adams PJ, Raeburn J, De Silva K (2009). A question of balance: prioritizing public health responses to harm from gambling. Addiction..

[4] Browne M, Greer N, Rawat V, Rockloff M (2017). A population-level metric for gambling-related harm. Int Gambl Stud.

[5] Currie SR, Hodgins DC, Wang J, El-Guebaly N, Wynne H, Chen S (2006). Risk of harm among gamblers in the general population as a function of level of participation in gambling activities. Addiction..

[6] Korn D, Gibbins R, Azmier J (2003). Framing public policy towards a public health paradigm for gambling. J Gambl Stud.

[7] Shaffer HJ, Kidman R, Grant J, Potenza M (2004). Gambling and the public health. Pathological gambling: a clinical guide to treatment.

[8] Wardle H, Reith G, Best D, McDaid D, Platt S. Measuring gambling-related harms: a framework for action. Gambling Commission 2018. https://www.gamblingcommission.gov.uk/PDF/Measuring-gambling-related-harms.pdf. Accessed 22 Apr 2020.

[9] Blaszczynski AP, McConaghy N (1989). The medical model of pathological gambling: current shortcomings. J Gambl Behav.

[10] American Psychiatric Association (2013). Diagnostic and statistical manual of mental disorders (DSM-5).

[11] Browne M, Greer N, Armstrong T, Doran C, Kinchin I, Langham E, Rockloff M. The social cost of gambling to Victoria: Victorian Responsible Gambling Foundation; 2017. https://responsiblegambling.vic.gov.au/resources/publications/the-social-cost-of-gambling-to-victoria-121/. Accessed 22 Apr 2020.

[12] Browne M, Rockloff MJ (2017). The dangers of conflating gambling-related harm with disordered gambling: commentary on: prevention paradox logic and problem gambling (Delfabbro & King, 2017). J Behav Addict.

[13] Delfabbro P, King D (2017). Prevention paradox logic and problem gambling: does low-risk gambling impose a greater burden of harm than high-risk gambling?. J Behav Addict.

[14] Delfabbro P, King DL (2019). Challenges in the conceptualisation and measurement of gambling-related harm. J Gambl Stud.

[15] King DL, Delfabbro PH (2018). The concept of “harm” in internet gaming disorder. J Behav Addict.

[16] Browne M, Langham E, Rawat V, Greer N, Li E, Rose J, et al. Assessing gambling-related harm in Victoria: a public health perspective: Victorian Responsible Gambling Foundation; 2016. https://responsiblegambling.vic.gov.au/resources/publications/assessing-gambling-related-harm-in-victoria-a-public-health-perspective-69/. Accessed 22 Apr 2020.

[17] Walker SE, Abbott MW, Gray RJ (2012). Knowledge, views and experiences of gambling and gambling-related harms in different ethnic and socio-economic groups in New Zealand. Aust N Z J Public Health.

[18] Ladouceur R (2004). Gambling: the hidden addiction. Can J Psychiatry.

[19] Browne M, Goodwin BC, Rockloff MJ (2018). Validation of the short gambling harm screen (SGHS): a tool for assessment of harms from gambling. J Gambl Stud.

[20] Browne M, Bellringer M, Greer N, Kolandai-Matchett K, Rawat V, Langham E, et al. Measuring the burden of gambling harm in New Zealand: New Zealand Ministry of Health; 2017. https://www.health.govt.nz/publication/measuring-burden-gambling-harm-new-zealand. Accessed 22 Apr 2020.

[21] Salomon JA, Vos T, Hogan DR, Gagnon M, Naghavi M, Mokdad A (2012). Common values in assessing health outcomes from disease and injury: disability weights measurement study for the global burden of disease study 2010. Lancet.

[22] Whiteford HA, Degenhardt L, Rehm J, Baxter AJ, Ferrari AJ, Erskine HE (2013). Global burden of disease attributable to mental and substance use disorders: findings from the global burden of disease study 2010. Lancet.

[23] Murray CJ, Lopez AD (2013). Measuring the global burden of disease. N Engl J Med.

[24] Blackman A, Browne M, Rockloff M, Hing N, Russell AM (2019). Contrasting effects of gambling consumption and gambling problems on subjective wellbeing. J Gambl Stud.

[25] Browne M, Rockloff MJ (2018). Prevalence of gambling-related harm provides evidence for the prevention paradox. J Behav Addict.

[26] Canale N, Vieno A, Griffiths MD (2016). The extent and distribution of gambling-related harms and the prevention paradox in a British population survey. J Behav Addict.

[27] Costa DS (2015). Reflective, causal, and composite indicators of quality of life: a conceptual or an empirical distinction?. Qual Life Res.

[28] Hodgins DC, Stea JN, Grant JE (2011). Gambling disorders. Lancet.

[29] Langham E, Thorne H, Browne M, Donaldson P, Rose J, Rockloff M (2015). Understanding gambling related harm: a proposed definition, conceptual framework, and taxonomy of harms. BMC Public Health.

[30] Callahan D (1973). The WHO definition of ‘health’. Hast Cent Stud.

[31] Browne M, Rockloff MJ (2019). Measuring behavioural dependence in gambling: a case for removing harmful consequences from the assessment of problem gambling pathology. J Gambl Stud.

[32] Ferris J, Wynne H. The Canadian problem gambling index: Canadian Centre on Substance Abuse; 2001. https://www.greo.ca/Modules/EvidenceCentre/files/Ferris%20et%20al(2001) The_Canadian_Problem_Gambling_Index.pdf. Accessed 22 Apr 2020.

[33] Shannon K, Anjoul F, Blaszczynski A (2017). Mapping the proportional distribution of gambling-related harms in a clinical and community sample. Int Gambl Stud.

[34] Williams RJ, Volberg R. Best practices in the population assessment of problem gambling: Ontario Problem Gambling Centre; 2010. https://opus.uleth.ca/bitstream/handle/10133/1259/2010-BP-OPGRC.pdf?sequence=1&isAllowed=y. Accessed 22 Apr 2020.

[35] Kind P, Lafata JE, Matuszewski K, Raisch D (2009). The use of QALYs in clinical and patient decision-making: issues and prospects. Value Health.

[36] Salomon JA, Haagsma JA, Davis A, de Noordhout CM, Polinder S, Havelaar AH (2015). Disability weights for the global burden of disease 2013 study. Lancet Glob Health.

[37] Schwarzinger M, Stouthard ME, Burström K, Nord E (2003). Cross-national agreement on disability weights: the European disability weights project. Popul Health Metrics.

[38] Browne M, Rawat V, Greer N, Langham E, Rockloff M, Hanley C (2017). What is the harm: scaling the PGSI to reflect the expected impact of gambling problems of quality of life. J Gambling Issues.

[39] Hilderink HB, Plasmans MH, Snijders BE, Boshuizen HC, Poos MR, van Gool CH (2016). Accounting for multimorbidity can affect the estimation of the burden of disease: a comparison of approaches. Arch Public Health.

[40] Mathers C, Vos T, Stevenson C, Begg S (2001). The burden of disease and injury in Australia. Bull World Health Organ.

[41] Flanagan W, McIntosh CN, Le Petit C, Berthelot JM (2006). Deriving utility scores for co-morbid conditions: a test of the multiplicative model for combining individual condition scores. Popul Health Metrics.

[42] James SL, Abate D, Abate KH, Abay SM, Abbafati C, Abbasi N (2018). Global, regional, and national incidence, prevalence, and years lived with disability for 354 diseases and injuries for 195 countries and territories, 1990–2017: a systematic analysis for the global burden of disease study 2017. Lancet.

[43] Mathers CD, Iburg KM, Begg S (2006). Adjusting for dependent comorbidity in the calculation of healthy life expectancy. Popul Health Metrics.

[44] Ferrari AJ, Stockings E, Khoo JP, Erskine HE, Degenhardt L, Vos T, Whiteford HA (2016). The prevalence and burden of bipolar disorder: findings from the global burden of disease study 2013. Bipolar Disord.

[45] Gadermann AM, Alonso J, Vilagut G, Zaslavsky AM, Kessler RC (2012). Comorbidity and disease burden in the National Comorbidity Survey Replication (NCS-R). Depress Anxiety.

[46] Doctor JN, Bleichrodt H, Lin HJ (2010). Health utility bias: a systematic review and meta-analytic evaluation. Med Decis Mak.

[47] Rehm J, Frick U (2010). Valuation of health states in the US study to establish disability weights: lessons from the literature. Int J Methods Psychiatr Res.

[48] Wiedermann W, Frick U (2014). Using surveys to calculate disability-adjusted life-years. Alcohol Res: Curr Rev.

[49] Yepes-Nuñez JJ, Zhang Y, Xie F, Alonso-Coello P, Selva A, Schünemann H, Guyatt G (2017). Forty-two systematic reviews generated 23 items for assessing the risk of bias in values and preferences' studies. J Clin Epidemiol.

[50] Boyd CM, Weiss CO, Halter J, Han KC, Ershler WB, Fried LP (2007). Framework for evaluating disease severity measures in older adults with comorbidity. J Gerontol Ser A Biol Med Sci.

[51] Petry NM, Stinson FS, Grant BF (2005). Comorbidity of DSM-IV pathological gambling and other psychiatric disorders: results from the National Epidemiologic Survey on alcohol and related conditions. J Clin Psychiatry.

[52] Brazier JE, Roberts J (2004). The estimation of a preference-based measure of health from the SF-12. Med Care.

[53] Morgan SL, Winship C (2015). Counterfactuals and causal inference.

[54] Rosenbaum PR, Rubin DB (1985). Constructing a control group using multivariate matched sampling methods that incorporate the propensity score. Am Stat.

[55] Lunceford JK, Davidian M (2004). Stratification and weighting via the propensity score in estimation of causal treatment effects: a comparative study. Stat Med.

[56] Li F, Morgan KL, Zaslavsky AM (2018). Balancing covariates via propensity score weighting. J Am Stat Assoc.

[57] Rockloff MJ, Browne M, Russell AM, Merkouris SS, Dowling NA (2019). A quantification of the net consumer surplus from gambling participation. J Gambl Stud.

[58] Abbott M, Binde P, Clark L, Hodgins D, Korn D, Pereira A, et al. Conceptual framework of harmful gambling: an international collaboration revised September 2015: Gambling Research Exchange Ontario; 2015. https://prism.ucalgary.ca/bitstream/handle/1880/51023/Conceptual_Framework_Oct_26_2015.pdf?sequence=1&isAllowed=y. Accessed 22 Apr 2020.

[59] Browne M, Hing N, Rockloff M, Russell AM, Greer N, Nicoll F, Smith G (2019). A multivariate evaluation of 25 proximal and distal risk-factors for gambling-related harm. J Clin Med.

[60] Cunha D, de Sousa B, Relvas AP (2017). Risk factors for pathological gambling along a continuum of severity: individual and relational variables. J Gambl Issu..

[61] Dowling NA, Merkouris SS, Greenwood CJ, Oldenhof E, Toumbourou JW, Youssef GJ (2017). Early risk and protective factors for problem gambling: a systematic review and meta-analysis of longitudinal studies. Clin Psychol Rev.

[62] Hing N, Russell A, Tolchard B, Nower L (2016). Risk factors for gambling problems: an analysis by gender. J Gambl Stud.

[63] Johansson A, Grant JE, Kim SW, Odlaug BL, Götestam KG (2009). Risk factors for problematic gambling: a critical literature review. J Gambl Stud.

[64] Miller H. Risk factors for problem gambling: environmental, geographic, social, cultural, demographic, socio-economic, family and household: Victorian Responsible Gambling Foundation; 2015. https://responsiblegambling.vic.gov.au/documents/22/risk-factors-for-problem-gambling.pdf. Accessed 22 Apr 2020.

[65] Sharpe L (2002). A reformulated cognitive–behavioral model of problem gambling: a biopsychosocial perspective. Clin Psychol Rev.

[66] Sharpe L, Tarrier N (1993). Towards a cognitive-behavioural theory of problem gambling. Br J Psychiatry.

[67] Vasiliadis SD, Jackson AC, Christensen D, Francis K (2013). Physical accessibility of gaming opportunity and its relationship to gaming involvement and problem gambling: a systematic review. J Gambl Issu..

[68] Williams RJ, West BL, Simpson RI. Prevention of problem gambling: a comprehensive review of the evidence and identified best practices: Ontario Problem Gambling Research Centre and Ontario Ministry of Health and Long Term Care; 2012. https://opus.uleth.ca/bitstream/handle/10133/3121/2012-PREVENTION-OPGRC.pdf?sequence=3&isAllowed=y. Accessed 22 Apr 2020.

[69] Lubman D, Manning V, Dowling N, Rodda S, Lee S, Garde E, et al. Problem gambling in people seeking treatment for mental illness: Victorian Responsible Gambling Foundation; 2017. https://responsiblegambling.vic.gov.au/resources/publications/problem-gambling-in-people-seeking-treatment-for-mental-illness-61/. Accessed 22 Apr 2020.

[70] Turner N, Ferentzy P. Review of problem gambling and comorbid disorders and behaviours: Final report. Ontario Problem Gambling Research Centre. 2012. https://www.greo.ca/Modules/EvidenceCentre/files/Turner%20et%20al(2012)Review_of_problem_gambling_and_comorbid_disorders_and_behaviours.pdf. Accessed 22 Apr 2020.

[71] Dowling NA, Cowlishaw S, Jackson AC, Merkouris SS, Francis KL, Christensen DR (2015). Prevalence of psychiatric co-morbidity in treatment-seeking problem gamblers: a systematic review and meta-analysis. Aust N Z J Psychiatry.

[72] Lorains FK, Cowlishaw S, Thomas SA (2011). Prevalence of comorbid disorders in problem and pathological gambling: systematic review and meta-analysis of population surveys. Addiction..

[73] Dowling NA, Cowlishaw S, Jackson AC, Merkouris SS, Francis KL, Christensen DR (2015). The prevalence of comorbid personality disorders in treatment-seeking problem gamblers: a systematic review and meta-analysis. J Personal Disord.

[74] Slade T, Johnston A, Oakley Browne MA, Andrews G, Whiteford H (2009). 2007 National Survey of mental health and wellbeing: methods and key findings. Aust N Z J Psychiatry.

[75] Grant BF, Hasin DS, Chou SP, Stinson FS, Dawson DA (2004). Nicotine dependence and psychiatric disorders in the United States: results from the national epidemiologic survey on alcohol and related conditions. Arch Gen Psychiatry.

[76] Hasin DS, Saha TD, Kerridge BT, Goldstein RB, Chou SP, Zhang H (2015). Prevalence of marijuana use disorders in the United States between 2001-2002 and 2012-2013. JAMA Psychiatry.

[77] Vos T, Mathers CD (2000). The burden of mental disorders: a comparison of methods between the Australian burden of disease studies and the global burden of disease study. Bull World Health Organ.

[78] Ruscio AM, Stein DJ, Chiu WT, Kessler RC (2010). The epidemiology of obsessive-compulsive disorder in the National Comorbidity Survey Replication. Mol Psychiatry.

[79] Goldstein RB, Smith SM, Chou SP, Saha TD, Jung J, Zhang H (2016). The epidemiology of DSM-5 posttraumatic stress disorder in the United States: results from the National Epidemiologic Survey on alcohol and related conditions-III. Soc Psychiatry Psychiatr Epidemiol.

[80] Kessler RC, Coccaro EF, Fava M, Jaeger S, Jin R, Walters E (2006). The prevalence and correlates of DSM-IV intermittent explosive disorder in the National Comorbidity Survey Replication. Arch Gen Psychiatry.

[81] Odlaug BL, Grant JE. Impulse-control disorders in a college sample: results from the self-administered Minnesota Impulse Disorders Interview (MIDI). Prim Care Companion J Clin Psychiatry. 2010;12(2). 10.4088/PCC.09m00842whi PMC2911005 20694115

[82] Andrews G, Henderson S, Hall W (2001). Prevalence, comorbidity, disability and service utilisation: overview of the Australian National Mental Health Survey. Br J Psychiatry.

[83] Haller H, Cramer H, Lauche R, Dobos G (2015). Somatoform disorders and medically unexplained symptoms in primary care: a systematic review and meta-analysis of prevalence. Dtsch Arztebl Int.

[84] Haagsma JA, De Noordhout CM, Polinder S, Vos T, Havelaar AH, Cassini A (2015). Assessing disability weights based on the responses of 30,660 people from four European countries. Popul Health Metrics.

[85] Casey P, Maracy M, Kelly BD, Lehtinen V, Ayuso-Mateos JL, Dalgard OS, Dowrick C (2006). Can adjustment disorder and depressive episode be distinguished? Results from ODIN. J Affect Disord.

[86] Kessler RC, Adler L, Barkley R, Biederman J, Conners CK, Demler O (2006). The prevalence and correlates of adult ADHD in the United States: results from the National Comorbidity Survey Replication. Am J Psychiatr.

[87] Winsper C, Bilgin A, Thompson A, Marwaha S, Chanen AM, Singh SP (2020). The prevalence of personality disorders in the community: a global systematic review and meta-analysis. Br J Psychiatry.

[88] van Schalkwyk MC, Cassidy R, McKee M, Petticrew M (2019). Gambling control: in support of a public health response to gambling. Lancet..

